# Influence of Risk of Drug–Drug Interactions and Time Availability on Patient Trust, Satisfaction, and Cooperation with Clinical Pharmacists

**DOI:** 10.3390/ijerph16091566

**Published:** 2019-05-05

**Authors:** Ying-Chyi Chou, Van Thac Dang, Hsin-Yi Yen, Kuan-Ming Lai

**Affiliations:** 1 Department of Business Administration, Tunghai University, Taichung 40704, Taiwan; ycchou@thu.edu.tw; 2 Department of Business Administration, Business School, Shantou University, Shantou 515063, China; 3 Department of International Business, Providence University, Taichung 43301, Taiwan; s8817007@mail2000.com.tw; 4 Department of Medical Administration, Taichung Veterans General Hospital, Taichung 40704, Taiwan; russ@vghtc.gov.tw

**Keywords:** risk of drug–drug interaction, trust, satisfaction, patient cooperation, clinical pharmacists

## Abstract

Patients with multiple diseases requiring several medications often face the risk of drug–drug interactions (DDIs). Such patients need more care and services from clinical pharmacists. Given the importance of this issue in clinical medicine, the present study aims to investigate how DDIs and time availability affect patient trust in clinical pharmacists and how patient trust influences patient satisfaction and cooperation between patients and clinical pharmacists. Sample data of 741 patients in central Taiwan hospitals were analyzed, and the results of structural equation modeling showed that DDIs and time availability positively affect patient trust, which, in turn, positively influenced patient satisfaction and cooperation between patients and clinical pharmacists. Overall, the results indicated that patient satisfaction is an important predictor of cooperation between patients and clinical pharmacists.

## 1. Introduction

Poor communication of drug therapy to patients often results in medication errors and adverse drug events. Clinical pharmacists play a vital role in managing the medication therapy of patients [1,2]. Well-trained clinical pharmacists often possess high levels of knowledge and skills that enable them to provide excellent pharmaceutical services to patients. However, some patients may not appreciate the professional services of clinical pharmacists because of a lack of trust [3,4].

The number of patients with multiple diseases requiring multiple medication therapies is increasing steadily [5]. When dealing with these patients, clinical pharmacists must combine several information sources to improve the accuracy of assessment of medication adherence [6]. Medication errors may occur in any part of the medication process and are affected by several factors, including the individual patient, the hospital physical environment, and medication-related factors [7]. To reduce the risk of polypharmacy, physicians and clinical pharmacists must collect information on patients’ visits to other medical institutions and the prescriptions received there [8]. As patients often lack the information and expertise needed to determine how to properly use various medications, they must completely place their trust in the expert knowledge of clinical pharmacists. Patients with multiple diseases who are at potential risk of drug–drug interactions (DDIs) often place their entire trust in clinical pharmacists. When a patient simultaneously suffers from multiple diseases, the probability of adverse drug reactions from improper or unnecessary medication is high and medical resources may be wasted. Rollason and Vogt [9] suggested that the participation of clinical pharmacists in medication counseling can reduce medication use and expenses. However, patients’ willingness to obey medication instructions depends on their level of trust in their clinical pharmacists. Despite the importance of this issue in clinical medicine, unfortunately, few studies have examined the relationship between DDI risk and patient trust in clinical pharmacists [10]. Thus, to provide empirical evidence and enrich the literature on clinical pharmacy, this study investigates the influence of DDI risk on patient trust in clinical pharmacists. 

Aparasu, Pharm, and Aparasu [1] stated that documenting the time of DDI occurrence, as well as understanding how clinical pharmacists manage and educate their patients, is critical. Clinical pharmacists often need time to communicate with their patients to obtain sufficient information and increase patient trust. Time availability (or communication time) may play a critical role in enhancing patient trust in clinical pharmacists. As such, this study also determines the communication time between patients and clinical pharmacists and how it affects patient trust, which, in turn, influences patient satisfaction and cooperation between patient and clinical pharmacist.

## 2. Literature Review

### 2.1. Drug–Drug Interaction (DDI) Risk and Trust in Clinical Pharmacists

DDIs are often a concern for patients and physicians as multiple medication use to manage complex diseases is becoming more common. Medicare beneficiaries and those prescribed with multiple medications may face the risk of receiving prescription combinations with potential DDIs [1]. Clinical pharmacists can play a critical role in managing the medication therapy of patients who are at risk for clinical DDIs. The knowledge of medication management services provided by clinical pharmacists is critical for patients who consume multiple medicines and are at an elevated risk of experiencing medication-related problems [11]. Clinical pharmacists are not only drug experts but also comprehensive pharmaceutical providers of care to patients. Therefore, the quality of the clinical pharmacist–patient relationship fundamentally depends on patient trust in clinical pharmacists [12]. Ngorsuraches [12] developed a scale called TRUST-Ph to measure patient trust in a community of clinical pharmacists. The measurement scale comprised three dimensions, namely benevolence, technical competence, and communication, and the Cronbach’s alpha values for these dimensions were 0.84, 0.86, and 0.91, respectively. This measurement scale was tested to have high reliability and validity and is widely used to examine patient trust. However, research examining antecedents of patient trust is lacking, and the role of DDI risk has not been discussed in previous clinical pharmacy literature. 

Trust is the degree of confidence that patients possess in their clinical pharmacist’s reliability and integrity. Trust is viewed as a comprehensive construct which reflects patient overall trust in clinical pharmacists. To address DDI risk, patients rely on the judgment of experts (i.e., clinical pharmacists) in professional systems. Clinical pharmacists can help patients manage their medicines and reduce their anxiety about taking multiple medicines. Although several factors affect patient trust, such as time of diagnosis, explanation, etc., DDI risk also affects patient trust. The reason for this is that when patients face the risk of DDI, they tend to rely on pharmacists to resolve this problem [11]. DDI risk raises patients’ anxiety and worry and facilitates patients to seek help from pharmacists. Because pharmacists can provide professional knowledge for patients, the patients will trust pharmacists and believe that pharmacists will help them to deal with DDI risk [12]. Thus, DDI risk may influence patient trust in pharmacists. However, once the trust relationship between a clinical pharmacist and a patient is broken, regaining the same is difficult. Previous research has demonstrated that patients who are at risk of experiencing medication-related problems express a lack of willingness to use the medication management services provided by clinical pharmacists [11]. Therefore, by providing expert knowledge and consultancy to patients, clinical pharmacists can help patients reduce their DDI risk and this in turn increases their trust in clinical pharmacists.

### 2.2. Time Availability and Trust in Clinical Pharmacists

Ahituv, Igbaria, and Sella [13] indicated that time pressure occurs when time is insufficient for an individual to seek a solution or to make an optimal decision. Stress can emerge when a person is asked to make a decision within a limited time. Janis [14] suggested that a person may not be able to consider every option when stress increases upon decision making. Time pressure regulates the amount of information that can be processed, and its effect on consumer decision making is significant [15]. Svenson, Edland, and Slovic [16] believed that time is the major factor affecting the quality of a decision. Payne, Bettman, and Johnson [17] also indicated that precision in exercising judgment decreases when time pressure increases because of insufficient time to manage all information. By constraining available time, time pressure will (1) restrict individuals’ ability to process information, (2) interfere with their ability to pay attention to the task, and (3) increase their effort to perform the task [18]. Heikki, Joseph, and Jeffrey [19] discovered that patients with comorbidities demonstrate lower confidence and that the patients’ confidence decreases when their time availability is reduced. When time availability is high, the quality of communication between clinical pharmacists and their patients is improved because clinical pharmacists have sufficient time to provide more information and instructions to patients. In contrast, if communication time is limited, clinical pharmacists may work under time pressure and be unable to provide personalized services. Thus, time availability indicates the quality of care that clinical pharmacists provide for their patients. Higher time availability represents a better quality of care and implies a higher level of patient trust in clinical pharmacists. 

### 2.3. Trust and Cooperation between Patient and Clinical Pharmacist

Caterinicchio [20] pointed out that patient trust is a feeling of confidence and reliance toward the physician. Anderson and Dedrick [21] considered patient trust as a belief and expectation that the physician will act to cure the illness. Dugan, Trachtenberg, and Hall [22] believed that patient trust is a belief that health care professionals have the patients’ best interests at heart, giving the latter the confidence to accept risk scenarios. Trust is defined as patient trust in medication instructions, clinical pharmacists’ knowledge, skills, consultancy, and their services. Clinical pharmacists often play pivotal roles in the continuum of health care provided to patients. Alberta College of Pharmacists [23] indicated that, when a patient doubts a clinical pharmacist’s professional ability, that patient’s subsequent behavior will be affected. Patient trust forecasts many medical procedure outcomes, such as whether the patient will follow medication instructions, as well as the quality of the medical relationship [24,25,26]. Therefore, the higher the level of trust in clinical pharmacists, the more likely it is that a patient will follow medication instructions and provide the clinical pharmacist detailed information on his or her disease condition. If a patient trusts in their clinical pharmacist, he or she is more willing to cooperate with the clinical pharmacist; for example, he or she will listen and follow the clinical pharmacist’s instructions, suggestions, and recommendations.

### 2.4. Trust and Satisfaction toward Clinical Pharmacists

Satisfaction is the customer’s positive perception of meeting or exceeding his or her economic and psychological expectations from the service providers [27]. Patients obtain satisfaction on the basis of how well clinical pharmacists meet their expectations. Thom, Hall, and Pawlson [25] pointed out that trust is a key factor that shapes these expectations. For example, Verbeek et al. [27] found a positive association between patient trust and their attitude toward physicians (β = 0.38, *p* < 0.01). Thom et al. [25] also found that physician accessibility has a strong association with patient trust in physicians (β = 0.39, *p* < 0.01). Trust is an important factor in the relationship between patients and physicians [28]. When patients trust their physicians, the former tend to be satisfied with the latter because of the belief that the physician will act in their best interests [22]. Pearson and Raeke [28] reported that patients who had difficulty obtaining referrals were more likely to report low trust in their primary care physician (adjusted odds ratio, 2.7; 95% confidence interval, 2.1–3.5). In contrast, patients reported higher satisfaction and trust in their physicians when they had easy access to healthcare services. 

### 2.5. Satisfaction and Cooperation between Patient and Clinical Pharmacist

Satisfaction is largely determined by the quality of customer experience and communication with the service providers [29]. Satisfaction influences customers’ outcomes, such as customer loyalty [30], customer commitment [31], and customer purchase intention [32]. According to social exchange theory, partners often demonstrate their commitment to relationships through their social investment in interaction and communication [29]. In the context of the clinical environment, when patients trust in and are satisfied with their clinical pharmacists, they are more likely to listen and follow the instructions of the latter. Patients are also more willing to cooperate with clinical pharmacists because they will obtain positive outcomes as a result of their cooperation. Eonnally III et al. [33] found that patients who were satisfied with physicians are more likely to follow their recommendations. Garman and colleagues [34] reported that satisfied patients are more likely to adhere to treatment recommendations and return for follow up consultations. De Paula et al. [35] suggested that patient satisfaction plays an important role in the interpersonal relationship between patient and therapist. This satisfaction will enhance patients’ attitude and cooperation with their therapist. Khomami [36] found a positive relationship between patient satisfaction and their willingness to cooperate with nurses. Conti and Humphris [37] indicated that patient satisfaction has a positive effect on the duration of the relationship between patients and physicians. According to prior studies, satisfaction may be a factor affecting patients’ willingness to cooperate with clinical pharmacists.

### 2.6. Research Questions

**Figure 1:**
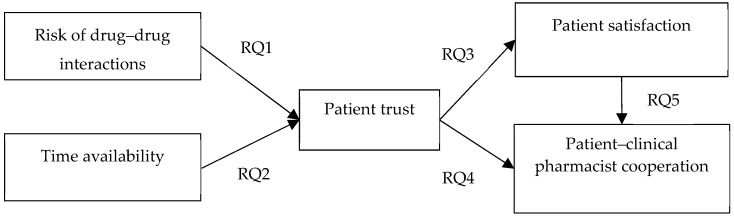
Research model.

Based on the literature review, a conceptual model is proposed in Figure 1. In this study model, answers to the following research questions (RQs) are examined:

RQ1: Does the risk of drug–drug interactions (DDIs) affect patient trust in clinical pharmacists?
RQ2: Does time availability (communication time) affect patient trust in clinical pharmacists?
RQ3: Does patient trust affect patient satisfaction toward clinical pharmacists?
RQ4: Does patient’ trust affect cooperation between patients and clinical pharmacists?
RQ5: Does patient satisfaction affect cooperation between patients and clinical pharmacists?

## 3. Methods

### 3.1. Sample and Data Collection

A list of patients who received medical care at central Taiwan hospitals was collected as the population. A computer generator was used to generate a random table of numbers, and a simple random sampling method was used to select respondents. A total of 748 patients were randomly selected as a sample and invited to complete a questionnaire. This questionnaire was designed using forward and backward translation from English to Chinese and vice versa. A pilot test was conducted with 16 patients to ensure the clarity and meanings of measurement items. After revision, the questionnaires were delivered to the selected participants. All questionnaires were returned, and 741 questionnaires were valid (response rate = 99.06%). 

Table 1 shows the demographic profiles of the respondents. Approximately 90.3% of the respondents were female, and only 9.7% were male. About 15.8% of the respondents were below 30 years old, 49% were between 31 and 40 years old, 30% were between 41 and 50 years old, and 5.3% were above 51 years old. About 78.1% of the respondents had a high school education or lower, 16.6% had a college education, and only 5.3% had a graduate education or higher. Approximately 93.9% of the respondents were married, and only 6.1% were not married. About 10.9% of the respondents had incomes below 20,000 NT, 64.8% had incomes between 20,000 and 40,000 NT, and 24.3% had incomes above 40,000 NT. About 66% of the respondents reported that they had been hospitalized previously, and about 34% reported that they had not.

### 3.2. Measures

To develop measures of variables in this study, a brainstorming method was applied with the participation of senior professional clinical pharmacists. Based on several discussions, a questionnaire was developed to investigate patients’ perceptions about their trust, satisfaction, and cooperation with clinical pharmacists. The questionnaire adopted a five-point Likert scale from 1 (completely disagree) to 5 (completely agree). Patient trust was measured using five items from Dugan et al. [22]. A sample item was “all in all, you trust physicians completely.” Patient satisfaction was measured using five items from Donnally III [33]. A sample item was “during the clinic visit, how would you rate your overall quality of care from the physician?” on a 5-point Likert scale of “excellent,” “very good,” “good,” “fair,” and “poor.” Cooperation with clinical pharmacists was measured using five items from Weingarten, Issa, and Posluszny [38]. A sample item was “I’m willing to cooperate with the physician.” Risk of DDI was measured using four items from Bosak et al. [39]. A sample item was “interactions between given medications might be life-threatening.” Time availability was measured using five items from Gendelman et al. [40] and Pozsgai et al. [41]. A sample item was “total time to diagnosis is long enough”.

### 3.3. Analytical Method

Statistical analysis was undertaken using IBM SPSS Statistics for Windows 20 and Amos 18. First, SPSS was used to analyze descriptive statistics and determine the reliability of the measures. Next, structural equation modeling with Amos 18 was used to test the validity and research questions in our study. 

## 4. Results

### 4.1. Descriptive Statistics

Table 2 shows the means, standard deviations, and Pearson correlations among variables in this study. The results indicated that risk of DDI is positively related to patient trust (*r* = 0.25, *p* < 0.01). Time availability was positively related to patient trust (*r* = 0.38, *p* < 0.01). Patient trust was positively related to patient satisfaction (*r* = 0.48, *p* < 0.01). Patient satisfaction was positively related to patient–clinical pharmacist cooperation (*r* = 0.56, *p* < 0.01). 

### 4.2. Confirmatory Factor Analysis

Confirmatory factor analysis was used to determine whether the hypothesized model fits the data well. The hypothesized model showed satisfactory goodness-of-fit indices: χ^2^/d.f. ratio was approximately 2.33 (χ^2^ = 97.86, d.f. = 42, *p* < 0.001), GFI = 0.97, CFI = 0.95, NFI = 0.96, TLI = 0.96, and RMSEA = 0.05. These results show that the goodness-of-fit measures for the hypothesized model meet the requirements for the benchmark fit indices (χ^2^/d.f. < 3, GFI > 0.90, CFI > 0.90, NFI > 0.90, TLI > 0.90, and RMSEA < 0.08) [42], thereby indicating that the conceptual model fits the data reasonably well. 

#### Reliability and Validity

Cronbach’s alpha was used to test the reliability of the measures [42]. The Cronbach’s α values for DDI risk, time availability, trust, satisfaction, and cooperation were 0.70, 0.70, 0.90, 0.80, and 0.90, respectively. The reliability of the whole measure was 0.96. Thus, the measurement scale of this study shows good reliability.

According to Kline [42], composite reliability (CR) and average variance extracted (AVE) are two indicators widely used to assess convergent validity. If the CR value exceeds 0.70 and the AVE value exceeds 0.50, convergent validity is satisfactory. The results show that convergent validity is sufficient in this study (DDI risk: CR = 0.92, AVE = 0.75; time availability: CR = 0.89, AVE = 0.63; patient trust: CR = 0.84, AVE = 0.52; patient satisfaction: CR = 0.86, AVE = 0.55; patient–clinical pharmacist cooperation: CR = 0.89, AVE = 0.63).

Discriminant validity is satisfactory if the square root of AVE is greater than the off-diagonal elements in the corresponding rows and columns of the Pearson correlation matrix [42]. As shown in Table 2, the square roots of AVE on the main diagonal are greater than those of the off-diagonal elements in the corresponding rows and columns of the matrix. Thus, the results evidently indicate good validity of the measure in this study.

As a self-reported survey was employed, common method bias was examined in this study. According to Podsakoff et al. [43], common method variance appears if the results of principal component analysis show a single factor emerging from an unrotated factor solution or if a first factor explains the majority of the variance. All items were subjected to principal component analysis, and the unrotated factor solution was examined. Five factors with an eigenvalue greater than 1.0 and which accounted for 70.13% of variance emerged. The first factor accounted for 29.89% of the variance. A single factor did not emerge, and the first factor did not explain the majority of the variance. Thus, common method variance does not seem to be a serious problem in our sample data.

### 4.3. Structural Equation Model

**Figure 2:**
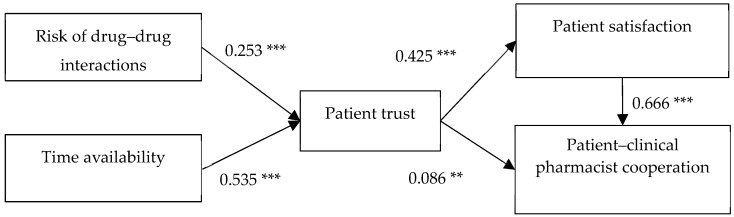
Results of structural equation modeling; (** *p* < 0.01, *** *p* < 0.001).

To provide further evidence to answer RQs 1 and 2, as well as to answer RQs 3 through 5, structural equation modeling was performed. As shown in Figure 2, the risk of DDI is significantly and positively related to patient trust (β = 0.253, *p* < 0.001), which supports RQ 1. Time availability is significantly and positively related to patient trust (β = 0.535, *p* < 0.01), which supports RQ 2. Patient trust is significantly and positively related to patient satisfaction (β = 0.425, *p* < 0.001), which supports RQ 3. Patient trust is significantly and positively related to patient–clinical pharmacist cooperation (β = 0.086, *p* < 0.01), which supports RQ 4. Finally, patient satisfaction is significantly and positively related to patient–clinical pharmacist cooperation (β = 0.666, *p* < 0.001), which supports RQ 5.

## 5. Discussion

This study aims to investigate the effect of DDI risk and time availability on patient trust, satisfaction, and cooperation between patients and clinical pharmacists. This study has several limitations. The data were collected only in hospitals in central Taiwan, which may limit the generalization of our findings. Future research should collect data from different areas and even countries to determine the validity of our findings. The use of a questionnaire to investigate patients’ perceptions presents its own limitations. Future research should adopt an experimental method to determine DDI risk. For example, the risk of some specific medicine interactions on certain types of patients may be determined.

This study shows several important findings. Firstly, DDI risk positively affects patient trust. This finding is consistent with findings by Aparasu et al. [1]. When patients use multiple medicines simultaneously, the DDI risk increases. Patients tend to trust their physicians because they believe physicians will provide useful suggestions and recommendations to reduce the DDI risk. 

Secondly, time availability is positively related to patient trust. This finding supports the notion that if physicians spend more time communicating with patients, they will provide more detailed information and better services for patients [13]. As a result, patients who receive better care may trust their physicians more because they believe physicians will act to bring the best outcomes for patients [15,40]. Furthermore, more time availability will enhance the quality of communication between patients and clinical pharmacists. Adequate communication time helps patients understand and follow the instructions of clinical pharmacists; thus, they are more likely to trust clinical pharmacists [41]. 

Thirdly, our results reveal that patient trust is positively related to patient satisfaction and cooperation between patients and clinical pharmacists. This result indicates that greater trust leads to higher satisfaction, that is, trust is an important predictor of patient satisfaction [24,25,26]. Furthermore, trust predicts cooperation between patients and clinical pharmacists. When patients trust their clinical pharmacists, they are more willing to cooperate with clinical pharmacists [28,33]. In addition, patient satisfaction is positively related to cooperation between patients and clinical pharmacists. This result shows the importance of patient satisfaction to the patients’ willingness to cooperate with their clinical pharmacists [38].

Finally, this study presents several implications for clinical pharmacists in dealing with and managing their patients. For patients with multiple diseases, DDI risk is often very high. These patients need more medication services from physicians. Thus, clinical pharmacists should spend more time on each patient. With sufficient time, clinical pharmacists can provide detailed information and careful instructions for their patients. Clinical pharmacists should exert greater effort to build patient trust because, when patients trust in clinical pharmacists, the former demonstrate higher levels of satisfaction and are more willing to cooperate with clinical pharmacists. These implications may improve the quality of medical treatments for patients.

## 6. Conclusions

The purpose of this study is to investigate the influence of risk of drug–drug interactions and time availability on patient trust, satisfaction, and cooperation with clinical pharmacists. Our findings show that DDI risk and time availability were positively related to patient trust. Furthermore, patient trust was positively associated with patient satisfaction and cooperation between patients and pharmacists. Finally, we found that patient satisfaction was positively related to cooperation between patients and pharmacists. Our findings provide empirical evidence for administrators and pharmacists in healthcare systems to understand and better manage their patients.

## Figures and Tables

**Table 1:** Demographic profiles of the respondents (*n* = 741).

Variables	Frequency	Percent
Gender		
Female	669	90.3%
Male	72	9.7%
Age		
Under 20	6	0.8%
21–30	111	15.0%
31–40	363	49.0%
41–50	222	30.0%
51 or above	39	5.3%
Education		
High school or below	579	78.1%
College or University	123	16.6%
Graduate or above	39	5.3%
Marital status		
Married	696	93.9%
Not married	45	6.1%
Income		
Under 20,000 NT	81	10.9%
20,000–under 40,000 NT	480	64.8%
40,000 NT or above	180	24.3%
Hospitalization or not		
Yes	489	66.0%
No	242	34.0%

**Table 2:** Means, standard deviations, and Pearson correlations.

Variable	Mean	SD	1	2	3	4	5
1. Risk of drug–drug interactions	4.05	0.58	0.96				
2. Time availability	4.03	0.55	0.17 **	0.94			
3. Patient trust	3.72	0.57	0.25 **	0.38 **	0.92		
4. Patient satisfaction	3.81	0.55	0.50 **	0.48 **	0.48 **	0.93	
5. Patient–clinical pharmacist cooperation	3.73	0.61	0.35 **	0.52 **	0.37 **	0.56 **	0.94

Note: *n* = 741; ** *p* < 0.01.
